# Glucocorticoid-dependence and independence of the circadian liver transcriptome

**DOI:** 10.1038/s44323-025-00068-8

**Published:** 2026-02-02

**Authors:** Robert J. Maidstone, A. Louise Hunter, Mudassar Iqbal, Nicola Begley, Matthew Baxter, Andrew S. I. Loudon, Magnus Rattray, David W. Ray

**Affiliations:** 1 NIHR Oxford Health Biomedical Research Centre, and NIHR Oxford Biomedical Research Centre, John Radcliffe Hospital, Oxford, UK; 2 Oxford Centre for Diabetes, Endocrinology and Metabolism, University of Oxford, Oxford, UK; 3 Oxford Kavli Centre for Nanoscience Discovery, University of Oxford, Oxford, UK; 4 Division of Diabetes, Endocrinology & Gastroenterology, School of Medical Sciences, Faculty of Biology, Medicine and Health, University of Manchester, Manchester, UK; 5 Centre for Biological Timing, Faculty of Biology, Medicine and Health, University of Manchester, Manchester, UK; 6 Division of Informatics, Imaging & Data Sciences, School of Health Sciences, Faculty of Biology, Medicine and Health, University of Manchester, Manchester, UK

**Keywords:** Circadian rhythms, Biochemistry

## Abstract

The liver, a key metabolic organ, shows clear day-night variation in gene and protein expression, and in metabolic pathway activity. Liver cells possess molecular circadian clocks, but systemic factors are critical contributors to the circadian rhythmicity of liver gene expression. Glucocorticoid action is reported to be one such factor. Glucocorticoids are essential hormones, with strong circadian oscillations, which control multiple cellular processes via the glucocorticoid receptor (GR). We compare clock factor and GR binding at promoters and enhancers linked to rhythmic genes and find increased clock factor binding at those promoter elements where GR is also present. Genes with GR binding at the promoter are more highly expressed and more likely to be detected as rhythmic. We then test the role of GR directly, by profiling rhythmic gene expression in mice with/without hepatocyte-targeted GR deletion. Notably, we observe only a small effect of GR deletion, with most rhythmic genes showing unchanged rhythmicity, despite evidence for local GR binding. We find a small number of genes showing lost or gained rhythmicity with GR deletion; genes which lose rhythmicity associate with GR binding sites. Contrary to expectations, GR appears redundant for conveying timing information to most of the rhythmic liver transcriptome.

## Introduction

Liver gene expression is robustly rhythmic over the day-night cycle, with studies reporting 16–40% transcripts showing a circadian rhythm of abundance in laboratory mice^1–4^. In the liver—perhaps more than in any other tissue—signals driven by the master suprachiasmatic nucleus clock, and the action of the local tissue clock converge to regulate gene expression^5,6^, although their relative contribution remains to be clarified^7^. 24 h variation in liver gene and protein expression, and indeed function, is of major importance to health, given the strong associations between hepatic/metabolic pathology (e.g. MASLD, T2DM) and circadian disruption (shift work, sleep disorders, mistimed meals)^8–10^. Factors conferring rhythmicity to the hepatic transcriptome have thus attracted considerable research interest.

Here, we examine the contribution of glucocorticoid signalling. Glucocorticoid hormones are essential for life, regulating energy metabolism and the inflammatory response. They are synthesised and secreted by the adrenal cortex in a strongly circadian manner, with superimposed responses to feeding and stress. The adrenal cortex is regulated both by sympathetic innervation and endocrine control in the form of pituitary ACTH. Circulating levels of glucocorticoid peak just before waking, which is thought to be important for the transition from the resting to the active phase. Glucocorticoid hormones act on the glucocorticoid receptor (GR, NR3C1), of the nuclear hormone receptor superfamily. In the liver, GR signalling is important for metabolism of glucose, lipids and amino acids^11^, and is directed by hepatic lineage determining factors including C/EBPβ^12^ and HNF4A^13^.

Multiple studies report interactions between GR and those transcription factors (TFs) that make up the molecular transcription-translation feedback loop of the circadian clock mechanism. Data suggest that clock TFs can serve to regulate and/or direct GR action. For example, CLOCK, one of the key TFs of the positive limb of the clock, can exert negative control over GR through acetylating its hinge region^14^. The Cryptochrome (CRY) proteins interact with multiple members of the nuclear receptor family, including GR, and have a repressive effect on GR action^15^. There is also evidence for a functional interaction between the circadian repressor REV-ERBα (NR1D1), also a nuclear receptor, and GR^16,17^. Conversely, GR and glucocorticoid actions can feed into the clock. When the existence of peripheral circadian oscillators was first reported, it became clear that glucocorticoid action was a powerful means to synchronise these oscillators^18^. This is thought to be through induction of Period (PER)^18^, which then feeds back to inhibit BMAL1/CLOCK, and thereby re-set the oscillator. In vivo, the actions of endogenous glucocorticoids are more subtle, with adrenalectomy serving to increase the rate of entrainment to phase-shifts in the light-dark cycle or feeding schedule^19,20^. Therefore, endogenous glucocorticoids may contribute to the stability of the central circadian oscillator and render it resistant to environmental change. Clarifying the contribution of glucocorticoid signalling to rhythmic liver function is important for understanding how disrupted adrenal glucocorticoid secretion might impact on liver physiology, and whether supraphysiological, and mistimed glucocorticoid exposure (as occurs with therapeutic glucocorticoid administration) might impact the liver circadian clock, and circadian energy metabolism.

Here, we interrogate the contribution of GR to the orchestration of the rhythmic liver transcriptome in vivo under circadian conditions (with no external light-dark cues). With in silico analyses of published datasets, we find that clock TF binding is greater at gene promoters where there is also GR binding. The majority of rhythmic genes in mouse liver have GR binding at the promoter, and we observe differences in distribution of phase between those rhythmic genes that do and do not have GR binding at the promoter. Genes that are GR-bound at the promoter are more likely to be rhythmic, even when we correct for the effect of expression level on likelihood of rhythmicity. We proceeded to test the contribution of GR by selectively deleting GR in the hepatocytes of adult mice, and profiling the circadian transcriptome. Whilst we find alterations in the expression pattern of a subset of core circadian clock genes, we find, remarkably, that rhythm is unchanged for the majority of cycling transcripts. A minority of transcripts lose or gain rhythmicity with GR knockout, findings which are replicated by a new analysis of existing time series liver gene expression data. Thus, GR is not required for the normal cycling of many circadian transcripts, and despite the high prevalence of GR binding in proximity to rhythmic genes, hepatocyte GR appears to play a minor role in conveying timing information to the liver transcriptome.

## Results

### GR and clock TF binding co-occur at gene promoters

**Fig. 1:**
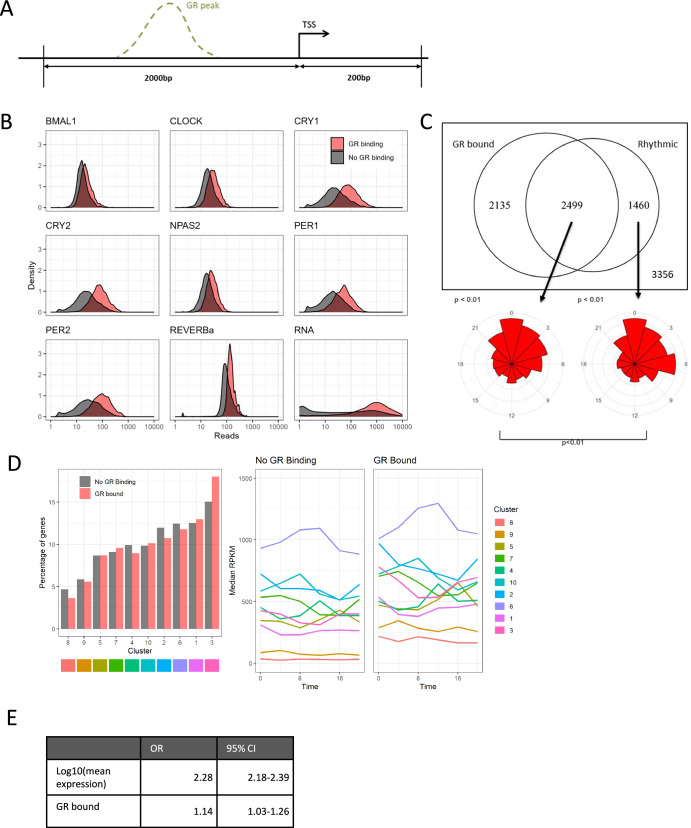
GR binding at the promoter is associated with the rhythmicity of gene expression. **A** Clock TF ChIP-seq reads were counted on promoter regions 2000bp downstream/400 bp upstream from the TSS. **B** Density plots of ChIP-seq reads (plus RNA-seq reads in bottom right plot) for GR bound promoters (red) and non-GR bound promoters (black) for clock TFs and gene expression. Clock TF ChIP-seq from Koike et al. (2012) with the exception of REVERBa from Hunter et al. (2020), RNA-seq data from Yang et al. (2016). Data averaged over multiple timepoints with exception of REVERBa where only a single timepoint (ZT8) was available. **C** Top: Venn diagram of genes bound by GR at the promoter and rhythmically expressed genes (JTK cycle, *p* < 0.05). Bottom: Peak phase of rhythmically called genes, both GR bound (left) and non-GR bound (right), *p*-values above plot are from a Rayleigh test of uniformity, *p*-value below the plot is from Watson’s test for homogeneity on two samples. **D** Cluster analysis on the dynamic behaviour of gene expression. Left: Percentage of GR and non-GR bound genes assigned to each cluster. Right: Median gene expression per cluster for GR and non-GR bound genes. **E** Odds ratios and 95% confidence interval from logistic regression model of rhythmicity (JTK-cycle defined) with log gene expression and presence of GR binding at promoter as covariates.

As previous work identified functional associations between GR and the core circadian transcription factors CLOCK, CRYs, and REV-ERBα^14–17^, we extended these observations to a more complete set of core clock TFs, based on genome binding distribution. We first studied all annotated promoter sequences, defined as −2000 bp, and +200 bp relative to the transcription start site (TSS) (Fig. 1A). We integrated liver GR ChIP-seq data^13^, from both vehicle- and dexamethasone-treated mice, with published clock factor ChIP-seq^21,22^ and RNA-seq data^23^ obtained from mouse liver under circadian conditions. We chose to use GR ChIP-seq data obtained from both vehicle- and dexamethasone-treated mice in order to identify as many potential GR binding sites as possible, acknowledging that this might identify sites seldom occupied under physiological conditions. GR binding at promoters was associated with increased binding of clock TFs, as indicated by increased mean ChIP-seq read counts over time (Fig. 1B), particularly in the case of CRY1/2 and PER1/2. We also observed that GR promoter-bound genes tended to be more highly expressed, as indicated by increased RNA-seq read counts (Fig. 1B; bottom right panel).

### GR promoter binding is associated with higher gene expression and increased likelihood of rhythmicity

We divided genes from the liver circadian RNA-seq series^23^ into those that did (JTK cycle; adjusted *p* < 0.05) and did not (adjusted *p* > 0.05) show rhythmic expression. We found that the majority (63%) of rhythmic genes had GR-bound promoters (Fig. 1C). Interestingly, we observed small differences in the distributions of phase between GR-bound and non-GR bound rhythmic genes, with two peak phases of gene expression evident in non-GR bound rhythmic genes (CT0, CT6), and a peak phase of expression at CT0 in GR-bound rhythmic genes (Fig. 1C). In nocturnal mice, the peak serum concentration of the endogenous GR ligand corticosterone is at the end of the inactive period (CT8-12)^24^, thus we anticipated that, if GR binding was driving rhythmic expression, we would see peak phase of GR-bound rhythmic genes at this time. By contrast, however, phase of GR-bound rhythmic genes was found to be nearly in antiphase to corticosterone levels, indicating that rhythmic control of these genes must be more complex.

As our counts-based analysis had indicated a difference in RNA expression levels between GR-bound and non-GR-bound genes (Fig. 1B*; bottom right panel*), we applied unsupervised clustering analysis to the circadian RNA-seq series (Fig. 1D*, left panel*), and plotted expression across time for each cluster (Fig. 1D, *right panel*). This approach supported the idea that GR-bound genes were more strongly expressed than non-GR-bound genes, and also raised the possibility that gene expression may be a significant confounder in rhythmicity analysis. Consequently, we analysed circadian rhythmicity as a function of logged gene expression, using a logistic regression model (Fig. 1E, Fig. S1A,B). We did indeed detect a highly significant relationship between mean expression level and likelihood of rhythmicity detection (Fig. 1E), but the odds ratio for rhythmicity for GR-bound genes remained >1, even when expression level was taken into account, thus indicating that GR binding was marking a set of circadian rhythmic genes irrespective of gene expression. In fact, a higher proportion of GR-bound genes were rhythmic, as compared to non-GR-bound genes, for non-extreme quantiles of expression (Fig. S1A).

GR-bound promoters may have more open chromatin which may in turn lead to increased ChIP-seq signal for other TFs (including our reported clock TFs). To check for this effect, we analysed the chromatin openness at our defined promoters using published DNase-seq data^25^. We found little difference in chromatin openness between GR-bound promoters and non-GR-bound promoters and still found an effect of GR binding on TF binding after adjusting for chromatin openness (Fig. S1C).

Taken together, these analyses suggest clock factor binding is greater at gene promoters where GR is also bound. Promoter GR binding is associated with a small but significant increase in likelihood of rhythmic gene expression, even when gene expression level is controlled for. Furthermore, rhythmic genes which are GR-bound show a different distribution of phase compared to rhythmic genes which are not GR-bound at the promoter. This supports the idea that, in mouse liver, GR binding at promoters may play a role in conferring rhythmicity to a subset of genes, but without a direct relationship between phase of the endogenous GR ligand, corticosterone, and phase of expression for GR-bound rhythmic genes.

### GR binds enhancers linked to rhythmic genes

**Fig. 2:**
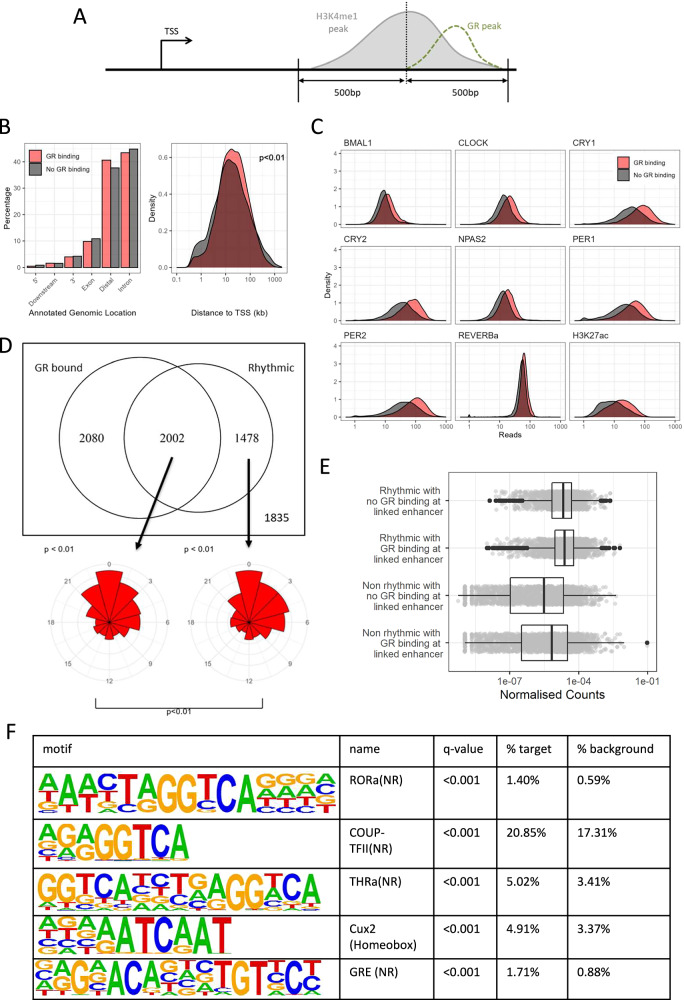
GR and clock factor binding co-occur at enhancers. **A** For enhancers defined by H3K4me1 peaks, clock TF ChIP-seq reads were counted on regions 500 bp either side of the consensus H3K4me1 peak. **B** Left: Annotated genomic locations of GR-bound (red) and non-GR-bound (black) enhancers. Right: Density plot of distance (in kb) from enhancer to closest transcription start site (TSS), p-value from a t-test on the logged data. **C** Density plots showing effect of GR binding (red) on clock TF binding (first 7 plots) and H3K27ac (8th plot) from Koike et al. and REVERBa (9th plot) Density plots showing amount of ChIP-seq reads for GR bound promoters (red) and non-GR-bound promoters (black) for clock TFs and H3K27ac. Clock TF and H3K27ac ChIP-seq from Koike et al. (2012) with the exception of REVERBa from Hunter et al. (2020). Data averaged over multiple timepoints with exception of REVERBa where only a single timepoint (ZT8) was available. **D** Top: Venn diagram of genes with linked enhancers (linked to nearest promoter) bound by GR and rhythmically expressed genes (JTK cycle, *p* < 0.05). Bottom: Peak phase of rhythmically called genes, both GR bound (left) and non-GR bound (right), p-values above plot are from a Rayleigh test of uniformity, p-value below the plot is from Watson’s test for homogeneity on two samples. **E** Gene expression (normalised counts) of genes with linked enhancers (each enhancer linked to closest gene TSS). Stratified by rhythmicity of gene and presence of GR binding at enhancer site. **F** Homer known motif analysis showing top 5 enriched motifs in GR-bound rhythmic enhancers as compared to a background of non-GR-bound rhythmic enhancers.

As GR binds to, and acts through distal regulatory elements, as well as promoters^26,27^, we extended our observations by studying GR binding at enhancers. We defined potential enhancers in mouse liver by calling peaks on H3K4me1 ChIP-seq data^22^ (Fig. 2A), a robust epigenetic marker for enhancers^28^. We excluded regions which overlapped with defined promoters (Fig. 1A). We again used our liver GR ChIP-seq data^13^, and integrated these with the published circadian TF binding atlas^22^ and liver REV-ERBα binding data^21^. We found that GR-bound and non-GR-bound enhancer elements showed a similar distribution across different types of genomic region (Fig. 2B*, left panel*), but GR-bound enhancers were more distal from transcription start sites than non-GR-bound enhancers (*p* < 0.01, Fig. 2B*, right panel*).

We then mapped ChIP-seq reads from the eight analysed clock TFs (BMAL1, CLOCK, CRY1, CRY2, PER1, PER2, NPAS2, and REV-ERBα), as well as the H3K27ac histone mark for transcriptionally-active chromatin, onto these enhancer regions (Fig. 2C), averaging over multiple timepoints. Here, we found evidence for increased binding of core circadian clock TFs at GR-bound enhancers. We also observed that the co-bound loci had a higher H3K27ac signature, suggesting a more active chromatin landscape (Fig. 2C).

To investigate potential functional consequences of co-bound enhancers, we linked all identified enhancers to their nearest gene promoter. We analysed rhythmicity of expression (JTK cycle adjusted *p* < 0.05) in genes linked to a GR-bound enhancer, and those linked to a non-GR-bound enhancer. As with GR-promoter binding, we found that the majority (58%) of circadian rhythmic genes were linked to a GR-bound enhancer (Fig. 2D). We once again observed a difference in phase distribution between rhythmic genes linked to GR-bound enhancers and those linked to a non-GR-bound enhancer (Watson’s test for homogeneity on two samples; *p* < 0.01), with a narrower distribution of phase evident in those rhythmic genes linked to a GR-bound enhancer, with peak phase of expression for both groups being CT0 (Fig. 2D).

For both rhythmic and non-rhythmic genes, those linked to GR-bound enhancers trended towards being more transcriptionally active compared to those linked to non-GR-bound enhancers, but this was not significant (Fig. 2E, *p* = 0.07). We performed motif analysis on enhancer sequences to identify possible TF binding motifs. Compared to a background of rhythmic enhancers not bound by GR, motif analysis of rhythmic GR-bound enhancers showed enrichment of GR binding sites (GRE), as well as other nuclear receptors which do not share similar binding sequences (ROR, COUP-TF, THRa) (Fig. 2F).

We again found a positive relationship between the likelihood of rhythmicity detection and expression level, but, in contrast to promoters, did not find increased likelihood of rhythmicity detection with enhancer GR binding (Fig. S2A–C), with the confidence interval for the odds ratio spanning 1.

We again considered the effect of chromatin openness on TF binding. Using published DNase-seq data^25^, we analysed openness at our defined enhancers. We found a slight increase in chromatin openness in GR-bound enhancers compared to non-GR-bound enhancers (Fig. S2D left). After adjusting for chromatin openness, we found that the increased clock TF binding in GR-bound enhancers was lost, suggesting that this was purely due to open chromatin effects (Fig. S2D right). Thus taken together, in contrast to what we observed at promoters, a contribution of GR-enhancer binding to rhythmicity is not clear.

As linking enhancers to the nearest gene does not confirm a functional link, we extended our analyses using published mouse liver promoter capture Hi-C data (pC-HiC)^29^ to link enhancers to genes. We found that 84% of the emergent genes were within the set we previously identified (Fig. S3A). Whilst the pC-HiC analyses were biased towards genes with higher expression (Fig. S3B) and linked enhancers tended to be more distal (Fig. S3C), it revealed a similar division by GR binding status, and rhythmicity detection (Fig. S3D) as our previous approach. After controlling for gene expression, we found that GR binding conferred a slight reduction in rhythmicity detection (Fig. S4A-C), potentially down to the bias towards genes with higher expression.

### Deletion of hepatocyte GR in adulthood remodels the liver transcriptome

Having found some associations in silico between GR binding, clock TF binding profiles, and rhythmic gene expression, we sought to test directly the contribution of GR to rhythmic liver gene expression by inducing GR deletion postnatally in the liver. We used the inducible, hepatocyte-targeted *Gr* (*Nr3c1*) knockout mice (*Nr3c1*^*flox/flox*^*Alb*^*CreERT2*^; GR-LKO) and littermate controls (*Nr3c1*^*flox/flox*^; WT) (Fig. S5A). We confirmed loss of liver GR protein expression with Western blot (Fig. S5B), following Cre induction with tamoxifen as previously described^21,30^.

**Fig. 3:**
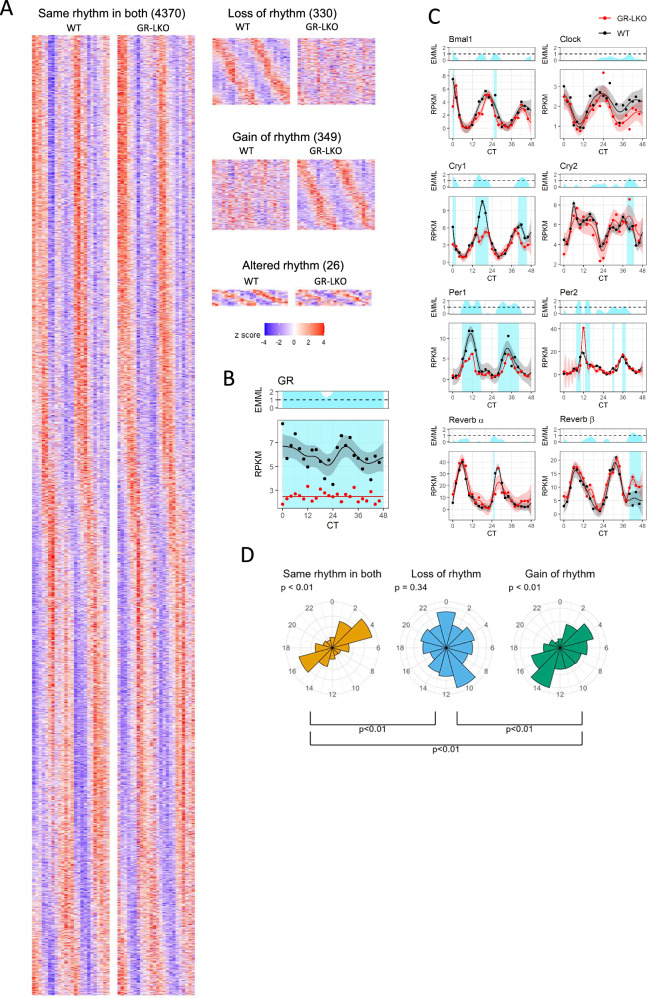
Hepatocyte GR deletion has a limited impact on the rhythmic liver transcriptome. **A** Heatmaps showing z-score normalised gene expression in the 4 rhythmic groups. Ordered by phase of gene (in relevant condition and WT in altered rhythm). **B** Time series of gene expression of GR (Nr3c1) for WT (black) and GR-LKO (red). Light blue shading shows regions of differential expression (EMML > 1). **C** Time series of gene expression of core clock genes for WT (black) and GR-LKO (red). Light blue shading shows regions of differential expression (EMML > 1). **D** Peak phase of genes by rhythmic group; same rhythm in both (phase of shared signal), gain of rhythm in GR-LKO (phase in GR-LKO) and loss of rhythm in GR-LKO (phase in WT), *p*-values above plot are from a Rayleigh test of uniformity, *p*-values below the plot are from Watson’s test for homogeneity on two samples.

We collected liver tissue from these mice every 2 h for 48 h, under circadian conditions (continuous dark; DD), and assessed liver gene expression by RNA-seq (Fig. 3A). As expected, GR gene expression was significantly reduced at all timepoints (Fig. 3B; EMML > 1, see “Methods”).

Differential gene expression was performed comparing WT mice against GR-LKO in all timepoints combined. We detected that 403 genes were differentially expressed; 197 up and 206 down in GR-LKO (Fig. S5C). In order to examine relationships between the GR cistrome, and altered gene expression we turned to peaks-to-genes analysis (PEGS^31^), using our GR ChIP-seq data^13^, and applying that to the differentially expressed genes. This analysis revealed that there was only statistically significant enrichment between GR binding and genes whose expression was reduced in the absence of GR (Fig. S5D), and therefore likely to be those genes positively regulated by glucocorticoids. In contrast, genes which were upregulated in the absence of GR only showed weak association with GR binding sites, supporting a distinctly different mechanism of gene regulation, and certainly compatible with an indirect mechanism of negative regulation requiring upregulation of an intermediary gene^13,32,33^.

### Rhythmic expression of many liver genes is independent of hepatocyte GR

We then proceeded to analyse differences in circadian rhythmicity of gene expression in the GR-LKO mice compared to WT. For this, we used the compareRhythms R package^34^. This is a rigorous approach for comparing circadian rhythmicity between conditions, and is an improvement over previous approaches which led to significant misclassification^34^. First, we filtered out genes which were not expressed at any timepoint in either WT or GR-LKO, or not expressed in both. With compareRhythms, genes were then classified as arrhythmic in both WT and GR-LKO (10,773 genes), showing the same rhythm in both WT and GR-LKO (4370 genes), showing a gain of rhythm in GR-LKO (349 genes), showing a loss of rhythm in GR-LKO (330 genes) or showing altered rhythm in GR-LKO (26 genes) (Fig. 3A). Additionally, 3836 genes did not meet the criteria for classification, indicating that rhythmic status could not be confidently ascribed.

The Period genes are highly glucocorticoid responsive, so are candidate mediators of GR actions on rhythmic liver gene expression. Therefore, in both the WT and GR-LKO samples, we assessed gene expression over time of the core circadian clock genes (*Bmal1, Clock, Cry1/2, Per1/2, Rev-erb α/β*) (Fig. 3C). Using a Gaussian process regression-based method^35^, we performed differential time-series analysis on the core clock genes. As expected, we saw changes in *Per1*, and *Per2* expression through time, and in *Cry1* at CT14-22 and again in the second cycle at CT40-46. However, analysis of the core circadian clock genes suggests the clock remained oscillatory in the GR-LKO livers, with all the components continuing to cycle, but with small differences in amplitude.

To investigate whether there might be a relationship between the phase of rhythmic gene expression and GR signalling, we plotted circadian time of peak expression (phase) for the rhythmic transcripts (Fig. 3D). Transcripts showing the same rhythm in WT and GR-LKO, and transcripts gaining rhythm in GR-LKO had clear patterns of phase distribution (Fig. 3D), but those transcripts losing rhythmicity in GR-LKO showed a much broader distribution, this was robust to the method of phase detection (replicated using JTK-cycle, Fig. S5E). This again was unexpected, as we might expect glucocorticoid-responsive genes to oscillate in phase with the endogenous ligand, corticosterone, but echoes what we observed with promoter GR-bound genes in our in silico analysis.

To investigate how the magnitude of gene expression impacted our rhythmicity analysis, we considered the “same rhythm in both” group, and compared amplitude with expression (Fig. S5F). As expected amplitude was highly correlated with expression in both WT and GR-LKO, but the relationship did not seem to differ between the two and differences between WT and GR-LKO amplitude and expression were also linearly correlated.

Taken together, our data show only modest differences in rhythmicity of liver gene expression between GR-LKO mice and WT. The vast majority of classified transcripts were arrhythmic in both genotypes, or showed the same rhythm in both genotypes (10,773 and 4370 genes, respectively), with only 705 genes showing some change in rhythmic expression (loss, gain, or alteration in rhythmicity). Phrased another way, 86% classified rhythmic genes showed the same rhythm in WT and GR-LKO samples. Overall, these observations support a limited role of hepatocyte GR in contributing to circadian rhythmicity of the liver transcriptome.

Previously published work on rhythmic gene expression in the liver, and the role of GR, suggested a much greater impact than we observed^36^. However, there are important differences between the studies, with the previous work conducted in the presence of light cycles (that is, not under circadian conditions, with light known to regulate the HPA axis and glucocorticoid production^37^), and employing different methods to compare rhythmicity between conditions. We took the opportunity to re-analyse the gene expression data from this earlier work using our pipeline, to permit direct comparisons between the studies (Fig. S6). In our new analysis of the published data, we observed that most rhythmic genes were unaffected by loss of the GR (Fig. S6A,B). With our approach, we noted a marked difference in the number of genes reported to lose rhythmicity with GR knockout compared to the original report (2980 vs 793). This reduced number is still greater than the 330 that we observed under strictly circadian conditions. We also saw a difference in the number of genes reported to show an increase in rhythmicity between the studies (fewer in our study). The circadian phase relationship also differed between the studies, possibly reflecting the effects of light cycles, with the largest group of rhythmic genes, those that remained rhythmic with and without GR, showing a peak phase at ZT 0-5. (Fig. S6B). Overall, when data from the two studies are analysed using a rigorous method for directly comparing rhythmicity, conclusions are similar. The majority of rhythmic genes show unchanged rhythmicity with loss of GR, with the larger numbers of genes that do change possibly reflecting the input of light cycles. For direct comparison, we also analysed gain and loss of rhythmicity using the JTK cycle method as used in the earlier publications^10^ (Fig. S6C). Here, a substantial difference in genes with ascribed changes in rhythmicity is seen (e.g. loss of rhythm with GR deletion = 933 genes with compareRhythms, and 2980 genes with JTK cycle analysis).

### Hepatocyte GR is required for the rhythmicity of a minor portion of the circadian transcriptome

**Fig. 4:**
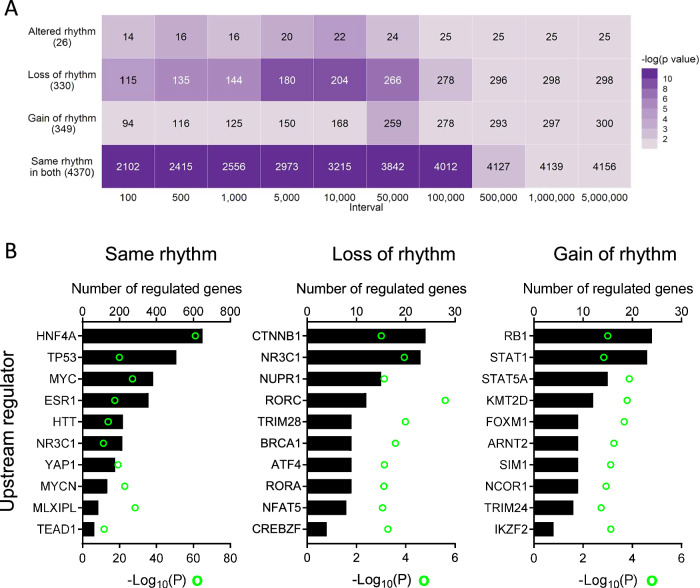
Direct regulation by GR is limited to a subset of the rhythmic liver transcriptome. **A** Peaks to genes analysis of enrichment of GR Chip-seq peaks in relation to distance from genes in rhythmic groups from compareRhythms analysis in WT and GR-LKO. **B** IPA upstream regulator analysis (transcription factors and nuclear receptors) for groups of rhythmic genes (altered rhythm group not analysed due to small size).

To gain insights into why certain genes did lose or gain rhythmicity with hepatocyte GR deletion, we examined these gene lists in greater detail. Pathway enrichment analysis (KEGG 2019 Mouse, WikiPathways 2019 Mouse, via Enrichr web portal) did not find any pathways to be significantly over-represented (adjusted *p* < 0.05) in any of the groups (data not shown). We did observe an enrichment of genes which lost rhythmicity in the GR-LKO mice in proximity to GR binding sites (PEGS analysis, Fig. 4A), which was not seen for genes which gained rhythmicity in GR-LKO. This suggests that GR does play a role in the direct regulation of these genes. However, enrichment was also seen, strongly, for those genes with the same rhythmicity of expression in GR-LKO and WT (Fig. 4A), further indicating that GR binding appears redundant for rhythmic expression of a large number of genes.

We performed upstream regulator analysis (IPA) for genes with the same, lost, or gained rhythmicity in GR-LKO (genes with altered rhythm were not further analysed, due to the very small number). Echoing the PEGS analysis, GR (NR3C1) was revealed as a putative upstream regulator for both genes in the same and loss of rhythm groups, but not for genes in the gain of rhythm group (Fig. 4B). The list of putative upstream regulators for the same rhythm group was notable for including established, broad-acting transcription factors such as HNF4A, TP53, MYC; HNF4A is of particular interest as it has been linked to definition of tissue circadian rhythms through BMAL1::CLOCK^38,39^. The emergence of MLXIPL (ChREBP) in this group, which is a nutrient-sensitive transcription factor, also indicates a mechanism through which timing of food intake (a potent zeitgeber to the liver^40,41^) could direct rhythmicity of gene expression. Thus, we can speculate that GR may play a role in regulating expression of this group of genes, but our circadian RNA-seq data suggests that it is redundant for the delivery of timing information, for which other TFs are dominant. For the loss of rhythm group, GR (NR3C1) was found as a putative upstream regulator alongside, interestingly, clock factors RORA and RORC. Although phase distribution was broad for this group of genes, peak phase of expression was CT10-12 (Fig. 3D), coinciding with the zenith of endogenous glucocorticoid levels. Therefore, GR likely does confer timing information to a proportion of the hepatic transcriptome, but what distinguishes this proportion is unclear based on current data.

For those genes which gain rhythmicity when GR is deleted, we observed the emergence of STAT1 and STAT5A as putative upstream regulators (Fig. 4B). There is a body of literature reporting interactions between the glucocorticoid receptor and both STAT1 and STAT5; for example, a direct interaction between GR and STAT5 in hepatocytes has been shown to regulate gene expression in mice^42^. Thus we can speculate that an outcome of GR-STAT interactions is that a proportion of genes are normally not rhythmic, but with GR removal, rhythmicity of expression emerges.

## Discussion

Glucocorticoid action in the liver has been strongly linked to the actions of the circadian clock, especially with regards to regulation of energy metabolism. Secretion of glucocorticoid hormones is both circadian and fasting-regulated. Excess glucocorticoid induces diabetes mellitus^43^. Previous work has identified functional crosstalk between the GR and core clock proteins, including CLOCK, CRY, and REV-ERBα^14–17^, and glucocorticoid actions in the liver are gated by circadian phase^17^. We set out to investigate GR-clock interaction further, first with in silico analysis of existing data, and then by testing directly the role of GR, in a postnatal, conditional, hepatocyte-targeted GR knockout mouse model.

We found that GR-bound promoters are significantly more likely to also be bound by core circadian clock transcription factors, including CLOCK, CRYs, REV-ERBα, and PERs. Our analysis also revealed that GR-promoter-bound genes are highly expressed in the liver, and we also found high gene expression to be an important confounder for the detection of rhythmicity. This is a predicted outcome and relates to the confidence of detecting a rhythmic signal over background noise increasing with the level of gene expression^22,34,44^. It also highlights the need to control for gene expression in comparing rhythmicity, to avoid confounding by expression level. Importantly, when we control for gene expression we still see significant association between promoter GR binding and circadian rhythmicity, with the majority of circadian rhythmic genes having GR-bound promoters. Interestingly, we discovered a significant difference in peak phase between GR-bound, and non-GR-bound promoters, implying that GR binding may be driving a distinct set of circadian targets.

We extended the analysis of GR and gene expression rhythmicity to enhancer elements, defined using the histone H3K4me1 mark. Again, most rhythmic genes had GR bound at a linked enhancer. The distribution of peak phase differed depending on whether GR was bound at the enhancer or not, but this effect was more subtle than observed in the promoter analysis. We also saw co-recruitment of a subset of circadian clock transcription factors, but this effect was lost once we controlled for chromatin openness in our analysis. Furthermore, when gene expression levels were taken into account, we did not see an increased likelihood of rhythmicity detection for genes with GR binding at linked enhancers. Therefore, we do not have evidence supporting a clear role for GR binding at enhancers in conferring rhythmicity to gene expression.

To investigate the causal role for GR in conferring rhythmicity to the liver we deleted GR in hepatocytes postnatally using an inducible Cre-Lox system. We conducted an experiment with frequent animal sampling over a full 48 h under true circadian conditions, constant darkness. We identified a high-confidence set of GR-dependent genes and mapped these to the known GR cistrome. As predicted, we found enrichment of the down-regulated genes for GR binding, but no relation between GR binding and the genes that were induced by loss of GR. This fits with the more robust and consistent mechanism of GR action as a transactivator. The genes which showed an increase in expression with GR loss may be regulated by a range of mechanisms, likely explaining the lack of a clear relationship with GR binding, a finding seen in other systems^13,32,33,45^.

We analysed gene expression through time, and compared between genotypes using compareRhythms. This approach directly compares rhythmicity between conditions and avoids misclassification resulting from categorisation of rhythmicity per condition, and the assumption that rhythmic changes can be inferred from differences in individual rhythmic status^34,46^. Interestingly, we found that similar numbers of genes gained and lost rhythms in the absence of GR, but that the majority of rhythmic genes showed unchanged rhythmicity. A gain or loss in rhythm maybe down to a complete loss/gain of rhythmicity or a change in the amplitude of the rhythm bringing it past the signal-to-noise ratio threshold needed to be detected.

We did observe small changes in circadian expression of several of the core clock genes, but this was clearly insufficient to affect the rhythmic status of the vast majority of rhythmic genes in intact animals. Here the preserved rhythmicity of the *Per* genes in the absence of GR, despite being glucocorticoid-responsive genes, likely reflects the sensitivity of *Per1/2* to other systemic cues (e.g. feeding time, insulin action^47,48^). The phase plots of the genes rhythmic in both genotypes showed clear peaks at CT4 and CT16. In contrast, the phase plots of genes which lost rhythm in the absence of GR had a broad distribution through the circadian cycle. This suggests that although GR is important for rhythmicity, it is not the primary signal conveying phase timing information, and are other zeitgebers which dominate, or can compensate for the loss of the GR signal. This finding resonates with earlier work looking at rhythmic changes in inflammatory status in lung which requires the presence of glucocorticoid, but does not rely on the glucocorticoid for timing information^24^. Similar finding are reported in intact zebrafish^49^.

Previous work had suggested that GR function in the liver may be important for setting the circadian landscape, with a more major impact on rhythmicity noted in liver GR null animals^36^. However, our work differs in some significant experimental, and analytical ways. We explored this further by re-analysing the previously published data using compareRhythms, and found our re-analysis to be far closer to our findings than in the original report, suggesting that some of the changes in rhythmic status previously reported may have resulted from categorisation issues. This is now a recognised issue with non-comparative rhythmicity analysis approaches (compareRhythms, and dryR). The re-analysed data did reveal interesting changes in phase, with those genes losing rhythm having a peak phase at ZT18, which does not align with the serum peak in corticosterone concentration (typically ZT10-12)^24^.

Our study focused on the convergence of GR and circadian clock action in the liver. Despite finding that most circadian genes were bound by GR, there was a surprisingly small impact on the circadian repertoire resulting from GR loss. The relatively small impact of GR loss on liver rhythmicity may be partly explained by the entraining influences present in an intact animal, including sympathetic innervation, and rhythmic feeding driven by the central clock. However, changes in liver gene rhythmicity are sensitive to physiological changes, and the enrichment of GR binding at rhythmic gene promoters suggested a functional role. Importantly, the genes that lost rhythmicity in the GR null animals were enriched in relation to GR binding sites, suggesting that these are genes which require GR action for rhythmicity, through a GR binding-dependent mechanism.

There are limitations to our work. We performed the computational analysis on previously published datasets, some of which were obtained years ago. The quality of data collected by different labs over time, and assembled for analysis may be variable, causing confounding, or bias, despite our best efforts. The observed strong association between GR binding and gene expression raised an important issue. This may have increased the risk of detecting spurious co-binding with clock TFs as a result of open and permissive chromatin, as indeed appeared to be the case at enhancers. We used arbitrary, but accepted, annotations to define promoters and enhancers in the early stages of the analysis using published data^21,22,50^. The hypotheses arising were then tested in conditional liver GR knockout animals. We defined circadian rhythmicity using well-accepted analytical algorithms, but we are well-aware of the risks of misclassification. We were careful to use a specific method to compare rhythmicity between conditions^4,34^. Our in-vivo analysis persisted through 48 h under circadian conditions. There was a relatively weak overlap between the identity of rhythmic genes in our analysis and those defined as rhythmic in earlier work. One issue is that the earlier work was not conducted under circadian conditions, and was over a single day-night cycle. However, analysis of both experimental datasets using the same method did, in both cases, detect that GR loss had a small effect on the proportion of rhythmic genes. Our upstream regulator analysis is computational, and brings in TFs which may not be relevant to the liver, but restricting analysis to only liver factors risks artificially constraining the analysis, and generating false negative results.

Taken together, our new data and analysis support a major presence of GR binding in the promoters. GR binding also is a major marker of high gene expression in the liver. However, under direct experimentation, the functional importance for GR in determining the circadian repertoire of the liver was clearly defined, but limited in scope. Therefore, in the liver, the actions of the GR are determined by the circadian clock to a greater extent than the actions of the circadian system are determined by the GR. This conclusion is strengthened by previous work examining the impact of adrenalectomy on circadian rhythmicity^20^. In this paper adrenalectomy had minimal impact on the orchestration of circadian rhythmicity. However, adrenalectomized animals re-entrained after feeding schedule phase shifts, implying a long feedback look involving rhythmic corticosterone may help stabilise the circadian clock in physiological settings, and render it resistance to shifting in response to environmental change.

It remains unclear what the functional importance of GR recruitment to highly expressed, and rhythmic genes in the liver is. It may be that more actively expressed genes, with open chromatin, are permissive for GR binding resulting in non-productive GR occupancy, or that the GR binding is rendered redundant by other TF recruitment. It is also possible that the role of the GR is unmasked under conditions of stress when GR is activated by ligand, although we did sample through two complete circadian cycles, and so through two cycles of circulating corticosterone oscillation, without seeing much effect. It may be that only a more severe insult to drive much higher than physiological corticosterone would be needed to see an effect, but this is would only be relevant under pathological conditions. As the importance of circadian rhythmicity becomes more widely acknowledged, it is important that studies adhere to robust analytical methodology to avoid false discovery^51^. In summary, in the liver, GR binding is a dominant feature of the circadian genome. However, we define only a small subset of rhythmic genes to be reliant on the GR for their circadian oscillation.

## Methods

All data were analysed in R (v4.0.2; R Core Team, 2020) using Rstudio (v2024.04.2-764^52^), unless otherwise mentioned, and plotted using ggplot2 (v3.5.1^53^) and patchwork (v1.2.0^54^).

### Animal experiments

The study protocol was approved by the University of Manchester Animal Welfare and Ethical Review Body, and carried out under project licence 70 8558 (Professor David A. Bechtold), according to the UK Animals (Scientific Procedures) Act 1986. Mice had ad libitum access to standard laboratory chow and water, and were group housed on 12 h:12 h light:dark (LD) cycles unless otherwise stated. Adult male and female mice were used in the study.

The *Nr3c1*^*flox/flox*^*Alb*^*CreERT2*^ line was created by crossing mice with the *Nr3c1*^*tm2Gsc*^ transgene^55^, kindly donated by Professor Jan Tuckermann (Universität Ulm, Germany), and mice with the *Alb*^*tm1(cre/ERT2)Mtz*^ transgene^30^ kindly donated by Professors Daniel Metzger and Pierre Chambon (IGBMC, Strasbourg, France), both on a C57BL/6 background.

Cre-mediated recombination was induced by injecting mice with 0.1 mg tamoxifen i.p. (T5648, Sigma-Aldrich) each day for 5 days, as previously described^21^. 14 days after the first injection, at ZT0, all mice were transferred to light-tight cabinets and maintained in constant darkness. Such a move from LD to constant dark conditions does not impair plasma corticosterone rhythms^56^ or the circadian rhythm of GR nuclear translocation^57^. After a further 24 h (CT0), tissue collection was commenced, with one Cre- (“WT”) and one Cre+ (“GR-LKO”) mouse being euthanised (by cervical dislocation) every 2 h for 48 h. As far as possible, the sex of the mice euthanised was alternated at each time point (e.g. males at CT0, CT4, CT8; females at CT2, CT6, CT10). Anaesthesia was not used.

### RNA extraction and sequencing

Total RNA was extracted from flash-frozen liver tissue using the ReliaPrep™ RNA Tissue Miniprep System (Promega), as per manufacturer instructions. Library preparation and RNA-sequencing using the HiSeq 4000 platform (Illumina) was carried out by the University of Manchester Genomic Technologies Core Facility as previously described^21^.

### Assessment of liver GR deletion

Liver GR deletion in tamoxifen-treated mice was confirmed by Western blot, using methods previously described^21^. The primary antibodies used were anti-GR (M-20) sc-1004 (Santa Cruz; RRID:AB_2155786), and anti-GAPDH 10494-1-AP (ProteinTech; RRID:AB_2263076), with DyLight 800 anti-rabbit secondary antibody 5151 P (Cell Signaling; RRID:AB_10697505). The blot was imaged using the LI-COR Odyssey system.

### Published clock TF and histone marker ChIP-seq data

Raw ChIP-seq data from ref. ^22^ (BMAL1, CLOCK, CRY1&2, NPAS2, PER1&2, H3K27ac and H3K4me1 from GEO accession number GSE39977) and ref. ^21^ (REV-ERBa from Array Express accession number E-MTAB-8413) was aligned to the mm10 genome using bowtie (v1.1.0^58^;) and sorted and indexed using Samtools (v1.2^59,60^).

Promoters were defined from the transcription start sites (TSS) (obtained from the R package TxDb.Mmusculus.UCSC.mm10.knownGene; v3.10.0^61^) with a promoter region being defined as −2000bp, and +200 bp relative to the TSS^50^.

Enhancers were defined by first calling peaks on H3K4me1 data using MACS (v2.1.1^62^). These peaks were removed if they intersected with the mm10 blacklist. Peaks from the five samples were then merged using DiffBind (v2.16.2^63^) and re-centred 500 bp either side of the median (across samples) summit; overlapping peaks were merged.

DiffBind was then used to count clock TFs and histone marks on these defined promoter and enhancer regions. Enhancers were linked to genes by finding the closest defined promoter. For clock TFs and H3K27ac from ref. ^22^ multiple timepoints (12 across 48 h) were counted onto promoter/enhancer regions separately and then means were taken. REV-ERBa data was only from ZT8.

Enhancer genomic locations were annotated using the annotatePeak function from the ChIPseeker R package (v1.24.0^64^) aligning to TxDb.Mmusculus.UCSC.mm10.knownGene (v3.10.0^61^).

Motif analysis was performed on GR-bound rhythmic enhancer regions as compared to a background of non-GR-bound rhythmic enhancers regions using Homer (v4.11.1^65^).

### Published circadian RNA-seq data

Normalised RNA-seq data from WT mice housed under LD conditions were obtained from ref. ^23^ (GEO accession number GSE70499).

Expression data were further normalised by read depth and then de-trended across each gene. Rhythm detection was performed using JTK-cycle (MetaCycle R package v1.2.0^66^). Rayleigh test of uniformity, used to determine if phases of genes were distributed uniformly, and Watson’s two-sample test of homogeneity, used to determine if two phase distributions were the same, were performed using the R package circular (v0.5-0^67^).

Gaussian mixture modelling was utilised to perform clustering on the dynamics of the gene expression data. First, the data was z-scored across time and then clustered using the R package Mclust (v6.1.1^68^).

To test for effect of GR binding on rhythmicity, whilst controlling for gene expression, JTK-rhythmicity classifications were modelled using a logistic regression model with log gene expression and presence of GR binding as covariates; glm function from stats R package (v4.0.2^69^). This was done using both continuous log gene expression and log gene expression binned into deciles.

### Published DNase-seq data

Normalised DNase-seq data from WT mouse liver housed under LD conditions were obtained from ref. ^25^ (GEO accession number GSE60430).

Data was lifted over from mm9 to mm10 using R package easyLift (v0.2.1^70^), before being counted onto defined promotor/enhancer regions using the R package PopSV (v.1.1^71^). The average value across time points (ZT2, 6, 10, 14, 18, 22) was taken.

To test for the effect of GR binding on core circadian clock TF binding, whilst controlling for chromatin openness, clock TF ChIP-seq reads were modelled by linear regression with DNase-seq reads for the same region and presence of GR binding as covariates. The R package effectsize (v1.0.1^72^;) was used to calculate standardised effect sizes and 95% confidence intervals for effect of GR binding.

### Published promoter-capture Hi-C data

Normalised promotor-capture Hi-C data from WT mouse liver housed under LD conditions was obtained from^29^ (https://github.com/mandok/circadian_3Dchrom).

Data was lifted over from mm9 to mm10 using R package easyLift (v0.2.1^70^;). Overlaps between interacting fragments and our defined enhancer regions were found using findOverlaps from the GenomicRanges R package (v1.58.0^50^).

### GR ChIP-seq

Mouse liver GR ChIP-seq was previously performed in C57BL/6 mice acutely treated with dexamethasone or cyclodextrin vehicle, with tissue collected one hour after drug administration^13^. That study utilised tissue collected at ZT6 (Zeitgeber Time 6; 6 h after lights on); for this study, we used both the ZT6 data and GR ChIP-seq data from tissue collected at ZT18 (18 hours after lights on) in the same experiment, to capture the GR cistrome across both day and night (ZT18 raw data available at GEO accession number GSE280340). The GR ChIP was performed as described in ref. ^13^, with ChIP libraries sequenced (paired-end) on the Illumina HiSeq 4000 platform. Reads were aligned to the mm10 genome using bowtie (v1.1.0^58^) and sorted and indexed using Samtools (v1.2^59,60^). Peaks were called across ZT6 and ZT18 samples, separately for dexamethasone- and vehicle-treated, using MACS (v2.1.1^62^), peaks were removed if they intersected with the mm10 blacklist.

Promoters/enhancers were called as “GR bound” if a GR peak overlapped with the promoter/enhancer region as defined above.

### WT and GR-LKO RNA-seq data

Adaptors were removed and ends trimmed using Trimmomatic (v0.36^73^;), then reads were mapped against the mouse genome (mm10/GRCm38) using STAR (v2.5.3^74^). Reads were counted, normalised and annotated using the Rsubread (v1.28.1^75^), edgeR (v3.30.3^76^) and biomaRt (v2.44.0^77^) packages, respectively.

Differential expression analysis was run in R using edgeR (v3.30.3^76^). Genes were considered to the differentially expressed (DE) if the false discovery rate (FDR) was less than 0.05. Peak set enrichment of gene sets was done using PEGS (v.0.3.0^31^). Differential rhythmicity analysis was performed on the RPKMs (calculated via edgeR) using the compareRhythms R package (v0.99.0^34,78^). A model selection approach was used with genes being assigned to either arrhythmic, differentially rhythmic (gain, loss or change of rhythm) or the same rhythm in both. A probability of being in a category of at least 0.6 was required for assignment. The “differentially rhythmic” category was then assigned gain, loss or change in rhythm based on which model had the highest probability to avoid losing genes that met the threshold for differential rhythmicity, but were unable to be distinguished between gain, loss or change of rhythm. Rayleigh test of uniformity, used to determine if phases of genes were distributed uniformly, and Watson’s two-sample test of homogeneity, used to determine if two phase distributions were the same, were performed using the R package circular (v0.5-0^67^). Differential time series analysis of the clock genes was performed using the R package nsgp (v1.0.5^35^). Upstream regulator analysis on rhythmic groups was done using QIAGEN IPA (QIAGEN Inc., https://digitalinsights.qiagen.com/IPA) (v111725566)^79^. Raw and processed RNA-seq data is available at GEO accession number GSE280339. The researcher conducting this data analysis was not blinded to the experimental groups, due to the nature of the analysis required.

For comparison, GR-LKO and WT RNA-seq data in LD mice from ref. ^36^ is analysed in the same way as our own data and presented.

## Supplementary information

Supplementalonly

ARRIVE E10 checklist - Maidstone et al

## Data Availability

All data are available to suitably qualified applicants. Genomic data are deposited for open access on GEO (RNA-seq at GEO accession number GSE280339 and ChIP-seq at GEO accession number GSE280340).

## References

[1] Zhang, R., Lahens, N. F., Ballance, H. I., Hughes, M. E. & Hogenesch, J. B. A circadian gene expression atlas in mammals: implications for biology and medicine. *Proc. Natl. Acad. Sci. USA.* **111**, 16219–16224 (2014). 25349387 10.1073/pnas.1408886111 PMC4234565

[2] Beytebiere, J. R. et al. Tissue-specific BMAL1 cistromes reveal that rhythmic transcription is associated with rhythmic enhancer-enhancer interactions. *Genes Dev* **33**, 294–309 (2019). 30804225 10.1101/gad.322198.118 PMC6411008

[3] Droin, C. et al. Space-time logic of liver gene expression at sub-lobular scale. *Nat. Metab.* **3**, 43–58 (2021). 33432202 10.1038/s42255-020-00323-1 PMC7116850

[4] Downton, P. et al. Chronic inflammatory arthritis drives systemic changes in circadian energy metabolism. *Proc. Natl. Acad. Sci. USA.* **119**, e2112781119 (2022). 35482925 10.1073/pnas.2112781119 PMC9170023

[5] Beytebiere, J. R., Greenwell, B. J., Sahasrabudhe, A. & Menet, J. S. Clock-controlled rhythmic transcription: is the clock enough and how does it work?. *Transcription* **10**, 212–221 (2019). 31595813 10.1080/21541264.2019.1673636 PMC6948975

[6] Kim, Y. H. & Lazar, M. A. Transcriptional control of circadian rhythms and metabolism: a matter of time and space. *Endocr. Rev.* **41**, 707–732 (2020). 32392281 10.1210/endrev/bnaa014 PMC7334005

[7] Hunter, A. L. & Bechtold, D. A. The metabolic significance of peripheral tissue clocks. *Commun. Biol.* **8**, 497 (2025). 40140664 10.1038/s42003-025-07932-0 PMC11947457

[8] Scheer, F. A. J. L., Hilton, M. F., Mantzoros, C. S. & Shea, S. A. Adverse metabolic and cardiovascular consequences of circadian misalignment. *Proc. Natl. Acad. Sci. USA* **106**, 4453–4458 (2009). 19255424 10.1073/pnas.0808180106 PMC2657421

[9] Vetter, C. et al. Night shift work, genetic risk, and type 2 diabetes in the UK Biobank. *Diabetes Care* **41**, 762–769 (2018). 29440150 10.2337/dc17-1933 PMC5860836

[10] Maidstone, R., Rutter, M. K., Marjot, T., Ray, D. W. and Baxter, M. Shift work and evening chronotype are associated with hepatic fat fraction and non-alcoholic fatty liver disease in 282,303 UK Biobank participants. *Endocr. Connect*. **13** (2024) 10.1530/EC-23-0472 PMC10831536 38055788

[11] Quagliarini, F., Makris, K., Friano, M. E. & Uhlenhaut, N. H. EJE Prize 2023: genes on steroids-genomic control of hepatic metabolism by the glucocorticoid receptor. *Eur. J. Endocrinol.* **188**, R111–R130 (2023). 37119521 10.1093/ejendo/lvad048

[12] Grøntved, L. et al. C/EBP maintains chromatin accessibility in liver and facilitates glucocorticoid receptor recruitment to steroid response elements. *EMBO J* **32**, 1568–1583 (2013). 23665916 10.1038/emboj.2013.106 PMC3671252

[13] Hunter, A. L. et al. HNF4A modulates glucocorticoid action in the liver. *Cell Rep* **39**, 110697 (2022). 35443180 10.1016/j.celrep.2022.110697 PMC9380254

[14] Nader, N., Chrousos, G. P. & Kino, T. Circadian rhythm transcription factor CLOCK regulates the transcriptional activity of the glucocorticoid receptor by acetylating its hinge region lysine cluster: potential physiological implications. *FASEB J.* **23**, 1572–1583 (2009). 19141540 10.1096/fj.08-117697 PMC2669420

[15] Lamia, K. A. et al. Cryptochromes mediate rhythmic repression of the glucocorticoid receptor. *Nature* **480**, 552–556 (2011). 22170608 10.1038/nature10700 PMC3245818

[16] Okabe, T. et al. REV-ERBα influences the stability and nuclear localization of the glucocorticoid receptor. *J. Cell Sci.* **129**, 4143–4154 (2016). 27686098 10.1242/jcs.190959 PMC5117207

[17] Caratti, G. et al. REVERBa couples the circadian clock to hepatic glucocorticoid action. *J. Clin. Investig.* **128**, 4454–4471 (2018). 30179226 10.1172/JCI96138 PMC6160001

[18] Balsalobre, A. et al. Resetting of circadian time in peripheral tissues by glucocorticoid signaling. *Science* **289**, 2344–2347 (2000). 11009419 10.1126/science.289.5488.2344

[19] Pezük, P., Mohawk, J. A., Wang, L. A. & Menaker, M. Glucocorticoids as entraining signals for peripheral circadian oscillators. *Endocrinology* **153**, 4775–4783 (2012). 22893723 10.1210/en.2012-1486 PMC3512018

[20] Le Minh, N., Damiola, F., Tronche, F., Schütz, G. & Schibler, U. Glucocorticoid hormones inhibit food-induced phase-shifting of peripheral circadian oscillators. *EMBO J.* **20**, 7128–7136 (2001). 11742989 10.1093/emboj/20.24.7128 PMC125339

[21] Hunter, A. L. et al. Nuclear receptor REVERBα is a state-dependent regulator of liver energy metabolism. *Proc. Natl. Acad. Sci. USA* **117**, 25869–25879 (2020). 32989157 10.1073/pnas.2005330117 PMC7568238

[22] Koike, N. et al. Transcriptional architecture and chromatin landscape of the core circadian clock in mammals. *Science* **338**, 349–354 (2012). 22936566 10.1126/science.1226339 PMC3694775

[23] Yang, G. et al. Timing of expression of the core clock gene Bmal1 influences its effects on aging and survival. *Sci. Transl. Med.* **8**, 324ra16 (2016). 26843191 10.1126/scitranslmed.aad3305 PMC4870001

[24] Ince, L. M. et al. Circadian variation in pulmonary inflammatory responses is independent of rhythmic glucocorticoid signaling in airway epithelial cells. *FASEB J.* **33**, 126–139 (2019). 29965797 10.1096/fj.201800026RR PMC6355062

[25] Sobel, J. A. et al. Transcriptional regulatory logic of the diurnal cycle in the mouse liver. *PLoS Biol*. **15** (2017) 10.1371/journal.pbio.2001069 PMC5393560 28414715

[26] Johnson, T. A., Paakinaho, V., Kim, S., Hager, G. L. and Presman, D. M. (2021) Genome-wide binding potential and regulatory activity of the glucocorticoid receptor’s monomeric and dimeric forms. *Nat. Commun*. **12** (1987). 10.1038/s41467-021-22234-9 PMC8012360 33790284

[27] McDowell, I. C. et al. Glucocorticoid receptor recruits to enhancers and drives activation by motif-directed binding. *Genome Res.* **28**, 1272–1284 (2018). 30097539 10.1101/gr.233346.117 PMC6120625

[28] Heintzman, N. D. et al. Histone modifications at human enhancers reflect global cell-type-specific gene expression. *Nature* **459**, 108–112 (2009). 19295514 10.1038/nature07829 PMC2910248

[29] Furlan-Magaril, M. et al. The global and promoter-centric 3D genome organization temporally resolved during a circadian cycle. *Genome Biol.* **22**, 162 (2021). 34099014 10.1186/s13059-021-02374-3 PMC8185950

[30] Schuler, M., Dierich, A., Chambon, P. & Metzger, D. Efficient temporally controlled targeted somatic mutagenesis in hepatocytes of the mouse. *Genesis* **39**, 167–172 (2004). 15282742 10.1002/gene.20039

[31] Briggs, P., Hunter, A. L., Yang, S.-H., Sharrocks, A. D. & Iqbal, M. PEGS: an efficient tool for gene set enrichment within defined sets of genomic intervals. *F1000Research* **10**, 570 (2021). 34504687 10.12688/f1000research.53926.1 PMC8406447

[32] Oh, K.-S. et al. Anti-inflammatory chromatinscape suggests alternative mechanisms of glucocorticoid receptor action. *Immunity* **47**, 298–309.e5 (2017). 28801231 10.1016/j.immuni.2017.07.012 PMC5572836

[33] Jubb, A. W., Young, R. S., Hume, D. A. & Bickmore, W. A. Enhancer turnover is associated with a divergent transcriptional response to glucocorticoid in mouse and human macrophages. *J. Immunol.* **196**, 813–822 (2016). 26663721 10.4049/jimmunol.1502009 PMC4707550

[34] Pelikan, A., Herzel, H., Kramer, A. & Ananthasubramaniam, B. Venn diagram analysis overestimates the extent of circadian rhythm reprogramming. *FEBS J.* **289**, 6605–6621 (2022). 34189845 10.1111/febs.16095

[35] Heinonen, M. et al. Detecting time periods of differential gene expression using Gaussian processes: an application to endothelial cells exposed to radiotherapy dose fraction. *Bioinformatics* **31**, 728–735 (2015). 25355790 10.1093/bioinformatics/btu699

[36] Quagliarini, F. et al. Cistromic reprogramming of the diurnal glucocorticoid hormone response by high-fat diet. *Mol. Cell* **76**, 531–545.e5 (2019). 31706703 10.1016/j.molcel.2019.10.007 PMC7928064

[37] Kalsbeek, A. et al. SCN outputs and the hypothalamic balance of life. *J. Biol. Rhythms* **21**, 458–469 (2006). 17107936 10.1177/0748730406293854

[38] Qu, M., Qu, H., Jia, Z. & Kay, S. A. HNF4A defines tissue-specific circadian rhythms by beaconing BMAL1::CLOCK chromatin binding and shaping the rhythmic chromatin landscape. *Nat. Commun.* **12**, 6350 (2021). 34732735 10.1038/s41467-021-26567-3 PMC8566521

[39] Qu, M., Duffy, T., Hirota, T. & Kay, S. A. Nuclear receptor HNF4A transrepresses CLOCK:BMAL1 and modulates tissue-specific circadian networks. *Proc. Natl. Acad. Sci. USA* **115**, E12305–E12312 (2018). 30530698 10.1073/pnas.1816411115 PMC6310821

[40] Damiola, F. et al. Restricted feeding uncouples circadian oscillators in peripheral tissues from the central pacemaker in the suprachiasmatic nucleus. *Genes Dev* **14**, 2950–2961 (2000). 11114885 10.1101/gad.183500 PMC317100

[41] Greenwell, B. J. et al. Rhythmic food intake drives rhythmic gene expression more potently than the hepatic circadian clock in mice. *Cell Rep.* **27**, 649–657.e5 (2019). 30995463 10.1016/j.celrep.2019.03.064

[42] Engblom, D. et al. Direct glucocorticoid receptor-Stat5 interaction in hepatocytes controls body size and maturation-related gene expression. *Genes Dev.* **21**, 1157–1162 (2007). 17504935 10.1101/gad.426007 PMC1865487

[43] Li, J.-X. & Cummins, C. L. Fresh insights into glucocorticoid-induced diabetes mellitus and new therapeutic directions. *Nat. Rev. Endocrinol.* **18**, 540–557 (2022). 35585199 10.1038/s41574-022-00683-6 PMC9116713

[44] Laloum, D. & Robinson-Rechavi, M. Methods detecting rhythmic gene expression are biologically relevant only for strong signal. *PLoS Comput. Biol.* **16**, e1007666 (2020). 32182235 10.1371/journal.pcbi.1007666 PMC7100990

[45] Auger, J.-P. et al. Metabolic rewiring promotes anti-inflammatory effects of glucocorticoids. *Nature* **629**, 184–192 (2024). 38600378 10.1038/s41586-024-07282-7

[46] Weger, B. D. et al. Systematic analysis of differential rhythmic liver gene expression mediated by the circadian clock and feeding rhythms. *Proc. Natl. Acad. Sci. USA.* **118**, e2015803118 (2021). 33452134 10.1073/pnas.2015803118 PMC7826335

[47] Hara, R. et al. Restricted feeding entrains liver clock without participation of the suprachiasmatic nucleus. Restricted feeding-induced Pergenes in the liver. *Genes Cells* **6**, 269–278 (2001). 11260270 10.1046/j.1365-2443.2001.00419.x

[48] Crosby, P. et al. Insulin/IGF-1 drives PERIOD synthesis to entrain circadian rhythms with feeding time. *Cell* **177**, 896–909.e20 (2019). 31030999 10.1016/j.cell.2019.02.017 PMC6506277

[49] Dickmeis, T. et al. Glucocorticoids play a key role in circadian cell cycle rhythms. *PLoS Biol* **5**, e78 (2007). 17373855 10.1371/journal.pbio.0050078 PMC1828142

[50] Lawrence, M., et al. Software for computing and annotating genomic ranges. *PLoS Comput. Biol*. **9** (2013) 10.1371/journal.pcbi.1003118 PMC3738458 23950696

[51] Ray, S. et al. Circadian rhythms in the absence of the clock gene Bmal1. *Science* **367**, 800–806 (2020). 32054765 10.1126/science.aaw7365

[52] Posit Software, PBC, Boston, MA RStudio: Integrated Development Environment for R (2024).

[53] Wickham, H. *Ggplot2: Elegant Graphics for Data Analysis* 2nd ed. (Springer International Publishing, 2016).

[54] Pedersen, T. L. Patchwork: The Composer of Plots (2024).

[55] Tronche, F. et al. Disruption of the glucocorticoid receptor gene in the nervous system results in reduced anxiety. *Nat. Genet.* **23**, 99–103 (1999). 10471508 10.1038/12703

[56] Oster, H. et al. The circadian rhythm of glucocorticoids is regulated by a gating mechanism residing in the adrenal cortical clock. *Cell Metab.* **4**, 163–173 (2006). 16890544 10.1016/j.cmet.2006.07.002

[57] Robles, M. S., Humphrey, S. J. & Mann, M. Phosphorylation is a central mechanism for circadian control of metabolism and physiology. *Cell Metab.* **25**, 118–127 (2017). 27818261 10.1016/j.cmet.2016.10.004

[58] Langmead, B., Trapnell, C., Pop, M. & Salzberg, S. L. Ultrafast and memory-efficient alignment of short DNA sequences to the human genome. *Genome Biol.* **10**, R25 (2009). 19261174 10.1186/gb-2009-10-3-r25 PMC2690996

[59] Danecek, P. et al. Twelve years of SAMtools and BCFtools. *Gigascience* **10** (2021) 10.1093/gigascience/giab008 PMC7931819 33590861

[60] Li, H. et al. The Sequence Alignment/Map format and SAMtools. *Bioinformatics* **25**, 2078–2079 (2009). and 1000 Genome Project Data Processing Subgroup. 19505943 10.1093/bioinformatics/btp352 PMC2723002

[61] Bioconductor Core Team, Bioconductor Package Maintainer *TxDb.Mmusculus.UCSC.mm10.knownGene: Annotation package for TxDb object(s*) (2019)

[62] Zhang, Y. et al. Model-based analysis of ChIP-Seq (MACS). *Genome Biol.* **9**, R137 (2008). 18798982 10.1186/gb-2008-9-9-r137 PMC2592715

[63] Stark, R. & Brown, G. D. DiffBind: differential binding analysis of ChIP-Seq peak data. *R package version*, **100** (2012)

[64] Yu, G., Wang, L.-G. & He, Q.-Y. ChIPseeker: an R/Bioconductor package for ChIP peak annotation, comparison and visualization. *Bioinformatics* **31**, 2382–2383 (2015). 25765347 10.1093/bioinformatics/btv145

[65] Heinz, S. et al. Simple combinations of lineage-determining transcription factors prime cis-regulatory elements required for macrophage and B cell identities. *Mol. Cell* **38**, 576–589 (2010). 20513432 10.1016/j.molcel.2010.05.004 PMC2898526

[66] Wu, G., Anafi, R. C., Hughes, M. E., Kornacker, K. & Hogenesch, J. B. MetaCycle: an integrated R package to evaluate periodicity in large-scale data. *Bioinformatics* **32**, 3351–3353 (2016). 27378304 10.1093/bioinformatics/btw405 PMC5079475

[67] Agostinelli, C., Lund, U. R package ‘circular’: Circular Statistics (2023)

[68] Scrucca, L., Fraley, C., Murphy, T. B. & Adrian, E., R. Model-based clustering, classification, and density estimation using mclust in R Chapman and Hall/CRC (2023).

[69] R. Core Team stats-package: The R Stats Package (2020).

[70] Lareau C. easyLift: convenience package for solving some liftover challenges_. R package version 0.2.1, https://github.com/caleblareau/easyLift (2017).

[71] Monlong J. PopSV: Population-based detection of structural variants from Read-Depth signal. R package version 1.1, https://github.com/jmonlong/PopSV (2015).

[72] Ben-Shachar, M., Lüdecke, D. & Makowski, D. effectsize: estimation of effect size indices and standardized parameters. *J. Open Source Softw.* **5**, 2815 (2020).

[73] Bolger, A. M., Lohse, M. & Usadel, B. Trimmomatic: a flexible trimmer for Illumina sequence data. *Bioinformatics* **30**, 2114–2120 (2014). 24695404 10.1093/bioinformatics/btu170 PMC4103590

[74] Dobin, A. et al. STAR: ultrafast universal RNA-seq aligner. *Bioinformatics* **29**, 15–21 (2013). 23104886 10.1093/bioinformatics/bts635 PMC3530905

[75] Liao, Y., Smyth, G. K. & Shi, W. The R package Rsubread is easier, faster, cheaper and better for alignment and quantification of RNA sequencing reads. *Nucleic Acids Res.* **47**, e47 (2019). 30783653 10.1093/nar/gkz114 PMC6486549

[76] Robinson, M. D., McCarthy, D. J. & Smyth, G. K. edgeR: a Bioconductor package for differential expression analysis of digital gene expression data. *Bioinformatics* **26**, 139–140 (2010). 19910308 10.1093/bioinformatics/btp616 PMC2796818

[77] Durinck, S., Spellman, P. T., Birney, E. & Huber, W. Mapping identifiers for the integration of genomic datasets with the R/Bioconductor package biomaRt. *Nat. Protoc.* **4**, 1184–1191 (2009). 19617889 10.1038/nprot.2009.97 PMC3159387

[78] Ananthasubramaniam, B. bharathananth/compareRhythms: compareRhythms for independently- and longitudinally- sampled time series data. Zenodo (2023).

[79] Krämer, A., Green, J., Pollard, J. Jr & Tugendreich, S. Causal analysis approaches in Ingenuity Pathway Analysis. *Bioinformatics* **30**, 523–530 (2014). 24336805 10.1093/bioinformatics/btt703 PMC3928520

