# Supplementary material for: Glucocorticoid-dependence and independence of the circadian liver transcriptome
Source: NPJ Biol Timing Sleep. 2026 Feb 2;3:6. doi: 10.1038/s44323-025-00068-8 (PMC12864027; doi:10.1038/s44323-025-00068-8)
Supplement: Supplementary file 1 — Supplementalonly [file 44323_2025_68_MOESM1_ESM.pdf]

A

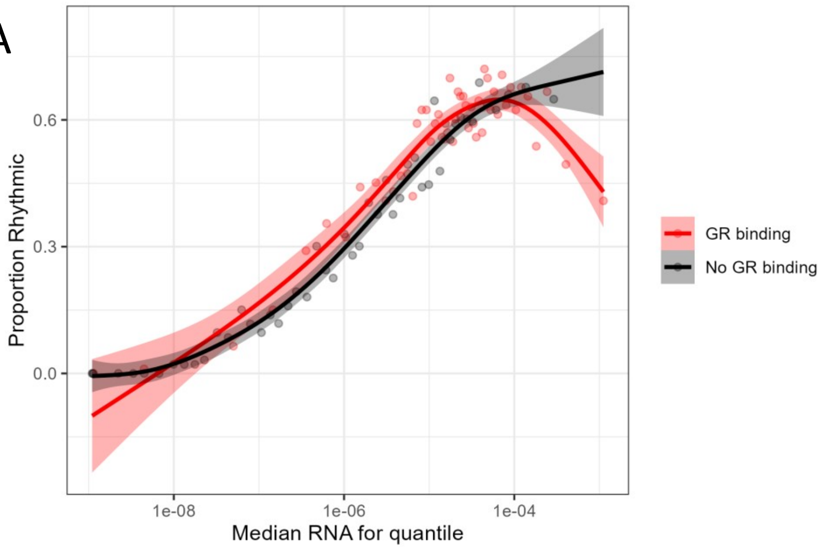

B

	OR	95% CI	# genes	# rhythmic genes	# genes with GR bound promoters
quantile 1	>100	0 - Inf	784	1	67
quantile 2	1.26	0.7 - 2.29	949	78	153
quantile 3	1.44	1.02 - 2.04	948	197	239
quantile 4	1.27	0.97 - 1.66	949	358	372
quantile 5	1.18	0.91 - 1.53	948	440	522
quantile 6	1.32	1.01 - 1.71	949	536	573
quantile 7	1.16	0.88 - 1.53	948	580	637
quantile 8	0.90	0.68 - 1.2	949	582	662
quantile 9	1.09	0.81 - 1.48	948	622	700
quantile 10	0.76	0.55 - 1.03	949	577	721

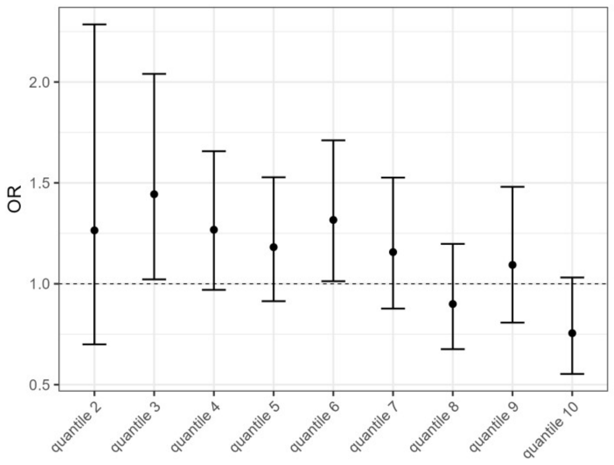

C

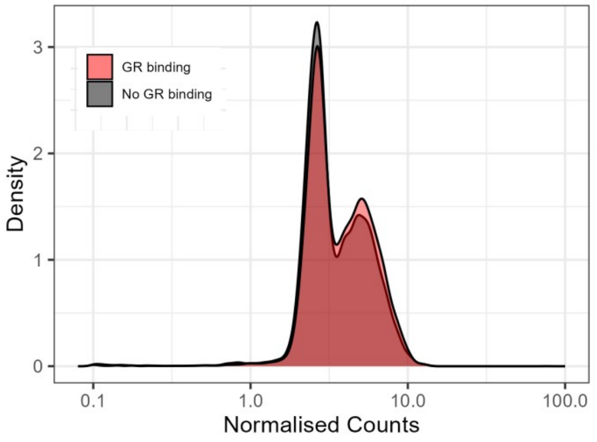

	Standardised Effect Size	95% CI
BMAL1	0.43	0.41 - 0.45
CLOCK	0.70	0.69 - 0.72
CRY1	0.71	0.69 - 0.72
CRY2	0.89	0.88 - 0.91
NPAS2	0.71	0.69 - 0.72
PER1	0.79	0.77 - 0.80
PER2	0.82	0.81 - 0.84
REVERBa	0.67	0.66 - 0.69

Figure S1. Effect of GR binding at promoters on gene rhythmicity is not modified by gene expression or chromatin openness.

A. For 50-quantiles of log gene expression, plot of median gene expression against proportion of rhythmic genes. Smoothed line and 95% CI (from generalised additive model with a cubic spline basis) to aid visualisation. **B.** Odds ratios and 95% confidence interval from logistic regression model of rhythmicity (JTK-cycle defined) with presence of GR binding at promoter as covariate. Stratified by deciles of log gene expression. Right: Forest plot of ORs and 95% CIs. **C.** Distribution of normalised DNase-seq read counts at promotor regions with and without GR binding. Table shows standardised effect size of GR binding on clock TF ChIP-seq reads adjusted by DNase-seq reads.

A

	OR	95% CI
Log10(mean expression)	2.05	1.95-2.15
GR bound	1.03	0.93-1.14

B

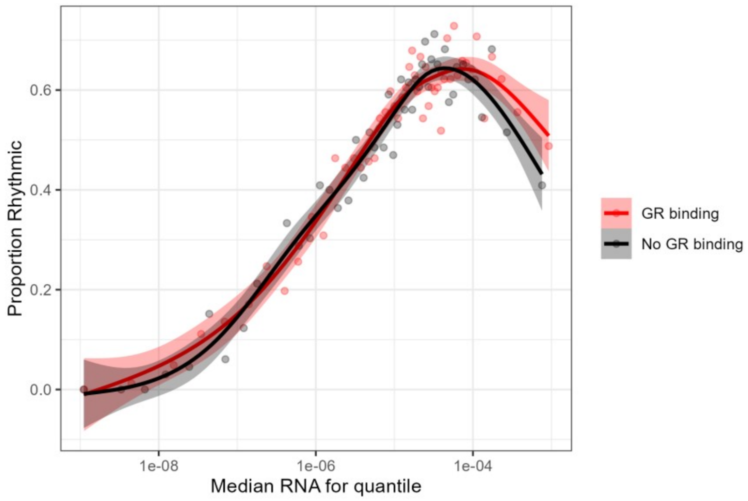

C

	OR	95% CI	# genes	# rhythmic genes	# genes with GR bound enhancers
quantile 1	1.78	0.72 - 4.4	673	20	311
quantile 2	0.95	0.66 - 1.37	743	143	361
quantile 3	1.03	0.76 - 1.39	743	272	387
quantile 4	0.99	0.74 - 1.32	744	356	394
quantile 5	1.12	0.83 - 1.49	743	412	404
quantile 6	1.17	0.86 - 1.58	743	458	442
quantile 7	0.77	0.57 - 1.04	744	467	424
quantile 8	0.84	0.62 - 1.14	743	462	441
quantile 9	1.26	0.93 - 1.7	743	479	443
quantile 10	1.20	0.89 - 1.63	744	430	462

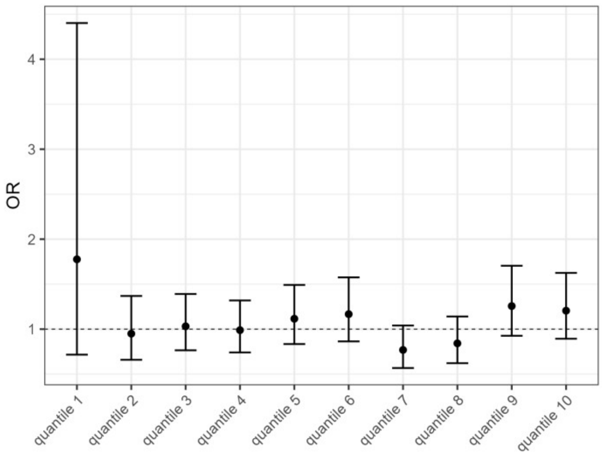

D

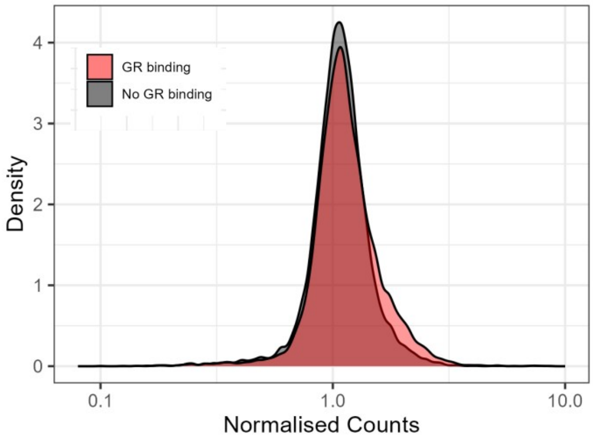

	Standardised Effect Size	95% CI
BMAL1	-0.05	-0.08 - -0.02
CLOCK	-0.05	-0.08 - -0.02
CRY1	-0.02	-0.05 – 0.00
CRY2	-0.03	-0.06 - -0.01
NPAS2	-0.05	-0.08 - -0.02
PER1	-0.04	-0.07 - -0.02
PER2	-0.03	-0.06 - -0.01
REVERBa	0.03	0.01 – 0.05

Figure S2. Effect of GR binding at enhancers on gene rhythmicity is modified by gene expression and chromatin openness.

A. Odds ratios and 95% confidence interval from logistic regression model of rhythmicity (JTK-cycle defined) with log gene expression and presence of GR binding at enhancer as covariates. **B.** For 50-quantiles of log gene expression, plot of median gene expression against proportion of rhythmic genes. Smoothed line and 95% CI (from generalised additive model with a cubic spline basis) to aid visualisation. **C.** Odds ratios and 95% confidence interval from logistic regression model of rhythmicity (JTK-cycle defined) with presence of GR binding at enhancer as covariate. Stratified by deciles of log gene expression. Right: Forest plot of ORs and 95% CIs. **D.** Distribution of normalised DNase-seq read counts at enhancers with and without GR binding. Table shows standardised effect size of GR binding on clock TF ChIP-seq reads adjusted by DNase-seq reads.

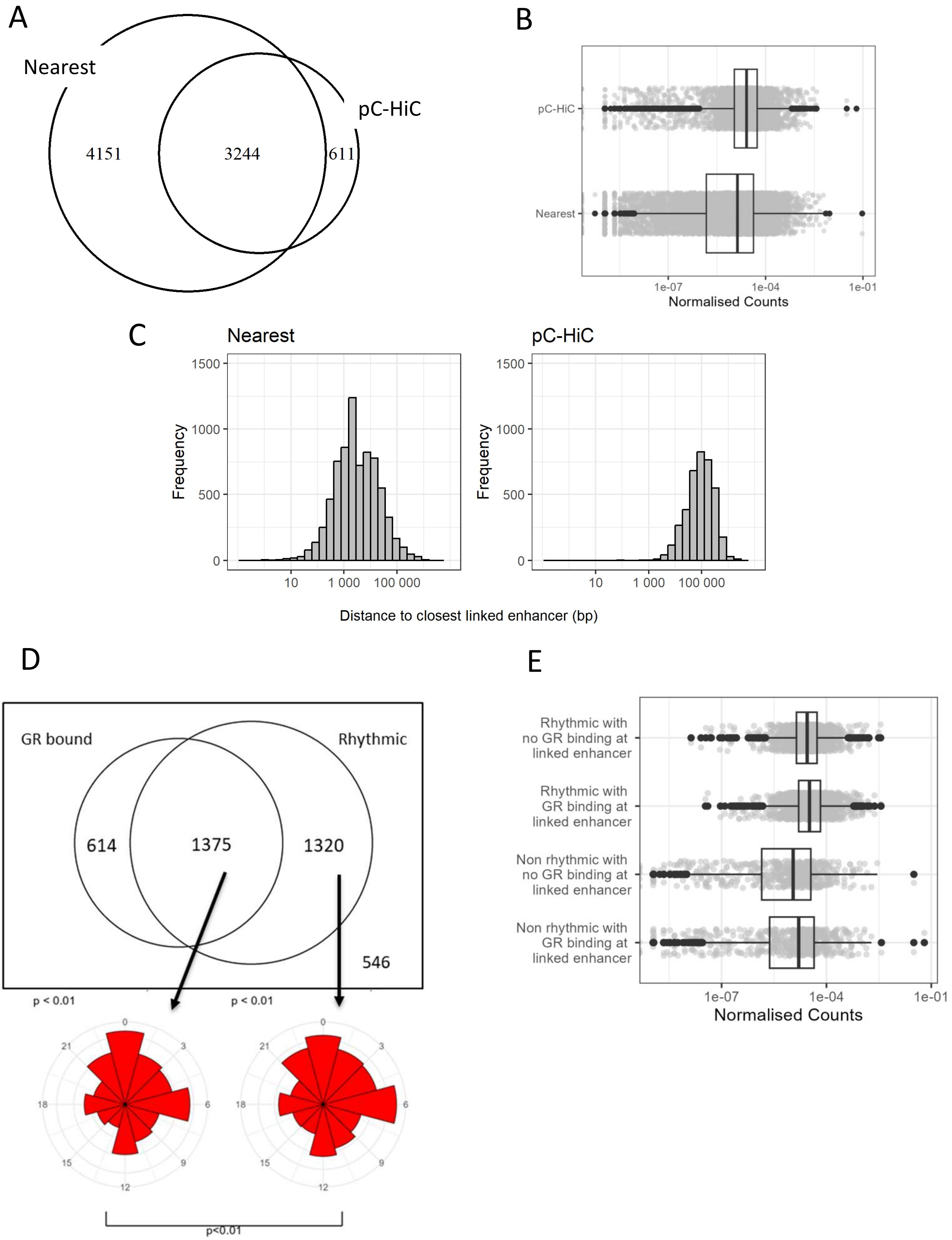
A
Nearest
pC-HiC
4151
3244
611
B
pC-HiC
Nearest
1e-07
1e-04
1e-01
Normalised Counts
C
Nearest
pC-HiC
1500
1000
500
0
Frequency
10
1 000
100 000
Distance to closest linked enhancer (bp)
D
GR bound
Rhythmic
614
1375
1320
546
p < 0.01
p < 0.01
0
3
6
9
12
15
18
21
p<0.01
E
Rhythmic with no GR binding at linked enhancer
Rhythmic with GR binding at linked enhancer
Non rhythmic with no GR binding at linked enhancer
Non rhythmic with GR binding at linked enhancer
1e-07
1e-04
1e-01
Normalised Counts

Figure S3. Comparison of using nearest enhancer to gene promotor to link with promoter capture Hi-C.

A. Overlap of genes with linked enhancer by nearest enhancer method (left) and promoter capture Hi-C (right). **B.** Gene expression (normalised counts) of genes with linked enhancers by nearest enhancer method and by promoter capture Hi-C. **C.** Comparison of distance to closest linked enhancer. **D-E.** Replication of previous results with enhancers linked to genes using promoter capture Hi-C. **D.** Top: Venn diagram of genes with linked enhancers bound by GR and rhythmically expressed genes (JTK cycle, $p<0.05$). Bottom: Peak phase of rhythmically called genes, both GR bound (left) and non-GR bound (right), p-values above plot are from a Rayleigh test of uniformity, p-value below the plot is from Watson’s test for homogeneity on two samples. **E.** Gene expression (normalised counts) of genes with linked enhancers. Stratified by rhythmicity of gene and presence of GR binding at enhancer site.

A

	OR	95% CI
Log10(mean expression)	1.46	1.43-1.50
GR bound	0.84	0.78-0.90

B

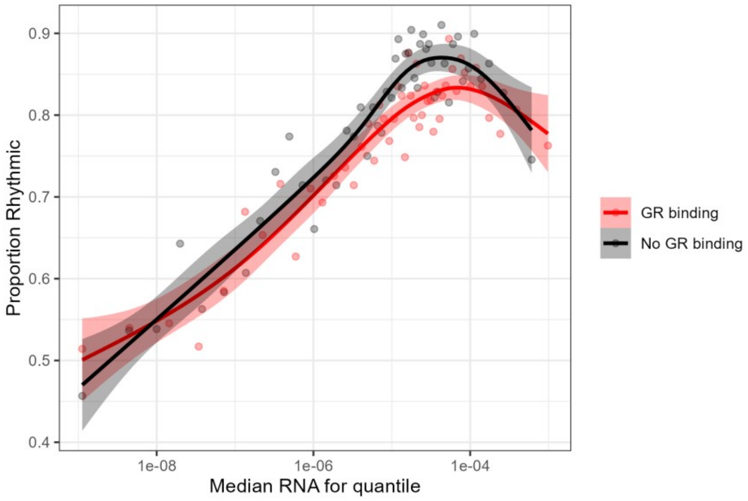

C

	OR	95% CI	# genes	# rhythmic genes	# genes with GR bound enhancers
quantile 1	0.93	0.76 - 1.13	1599	861	744
quantile 2	0.94	0.77 - 1.15	1735	1160	830
quantile 3	0.91	0.74 - 1.13	1735	1252	852
quantile 4	0.95	0.76 - 1.2	1735	1357	837
quantile 5	0.7	0.54 - 0.89	1736	1426	863
quantile 6	0.77	0.59 - 1.01	1735	1475	893
quantile 7	0.52	0.4 - 0.68	1733	1461	903
quantile 8	0.9	0.69 - 1.16	1735	1456	949
quantile 9	0.84	0.64 - 1.11	1735	1491	940
quantile 10	0.84	0.65 - 1.07	1735	1411	994

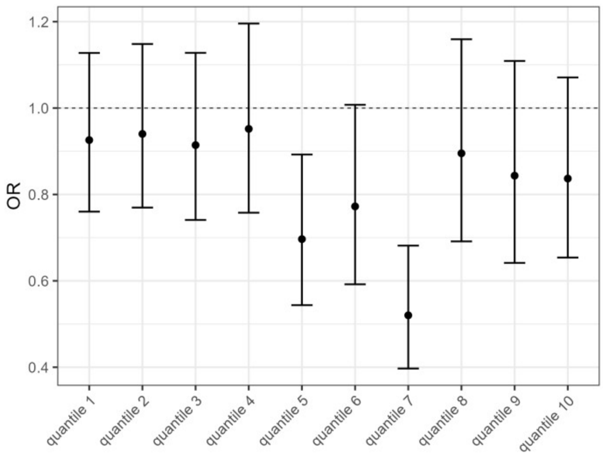

Figure S4. Effect of GR binding at enhancers on gene rhythmicity is still modified by gene expression when replicated with enhancers linked to genes using promoter capture Hi-C.

A. Odds ratios and 95% confidence interval from logistic regression model of rhythmicity (JTK-cycle defined) with log gene expression and presence of GR binding at enhancer as covariates. **B.** For 50-quantiles of log gene expression, plot of median gene expression against proportion of rhythmic genes. Smoothed line and 95% CI (from generalised additive model with a cubic spline basis) to aid visualisation. **C.** Odds ratios and 95% confidence interval from logistic regression model of rhythmicity (JTK-cycle defined) with presence of GR binding at enhancer as covariate. Stratified by deciles of log gene expression. Right: Forest plot of ORs and 95% CIs.

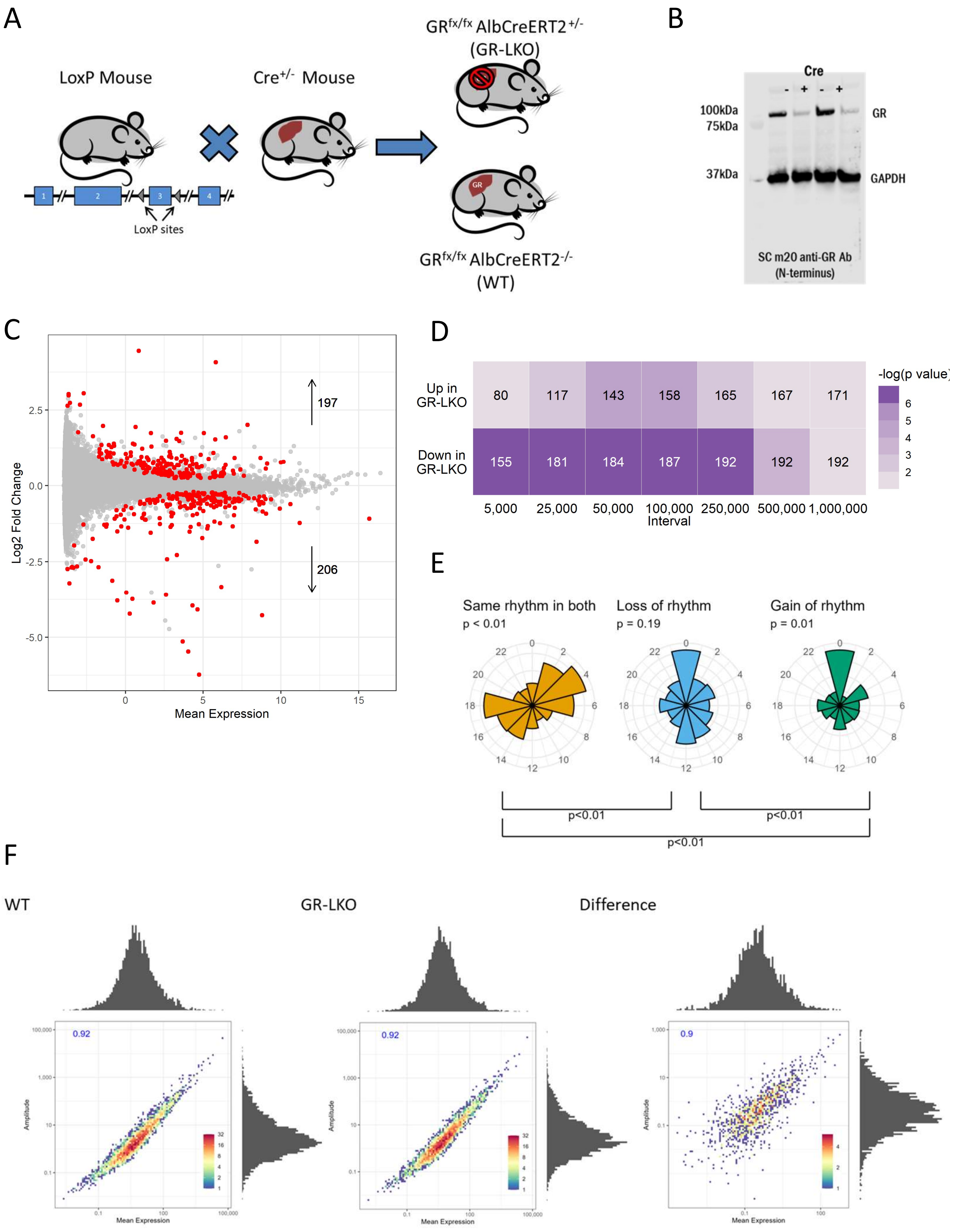
A
LoxP Mouse
Cre+/- Mouse
LoxP sites
GRfx/fx AlbCreERT2+/-
(GR-LKO)
GRfx/fx AlbCreERT2-/-
(WT)
GR
B
Cre
- + - +
100kDa
75kDa
37kDa
GR
GAPDH
SC m20 anti-GR Ab
(N-terminus)
C
Log2 Fold Change
Mean Expression
197
206
D
Up in GR-LKO
Down in GR-LKO
80 117 143 158 165 167 171
155 181 184 187 192 192 192
5,000 25,000 50,000 100,000 250,000 500,000 1,000,000
Interval
-log(p value)
E
Same rhythm in both
p < 0.01
Loss of rhythm
p = 0.19
Gain of rhythm
p = 0.01
p<0.01
p<0.01
p<0.01
F
WT
GR-LKO
Difference
0.92
0.92
0.9
Amplitude
Mean Expression

Figure S5. Hepatocyte GR deletion impacts the liver transcriptome.

A. Cartoon showing breeding of GR liver specific knockout mouse. **B.** Western blot of GR and GAPDH (as a control) proteins for Cre +/- mice. **C.** MA plot of differential gene expression of GR-LKO as compared to WT. **D.** Peaks to genes analysis of enrichment of GR Chip-seq peaks in relation to distance from differentially expressed genes in GR-LKO. **E.** Peak phase of genes by rhythmic group; same rhythm in both (phase of shared signal), gain of rhythm in GR-LKO (phase in GR-LKO) and loss of rhythm in GR-LKO (phase in WT). Phases calculated using JTK-cycle, groups from compareRhythms. p-values above plot are from a Rayleigh test of uniformity, p-values below the plot is from Watson's test for homogeneity on two samples. **F.** Correlation of amplitude and mean expression of genes with "same" rhythm in both WT and GR-LKO as determined using compareRhythms. Analysis done in both WT and GR-LKO as well as on differences between the two.

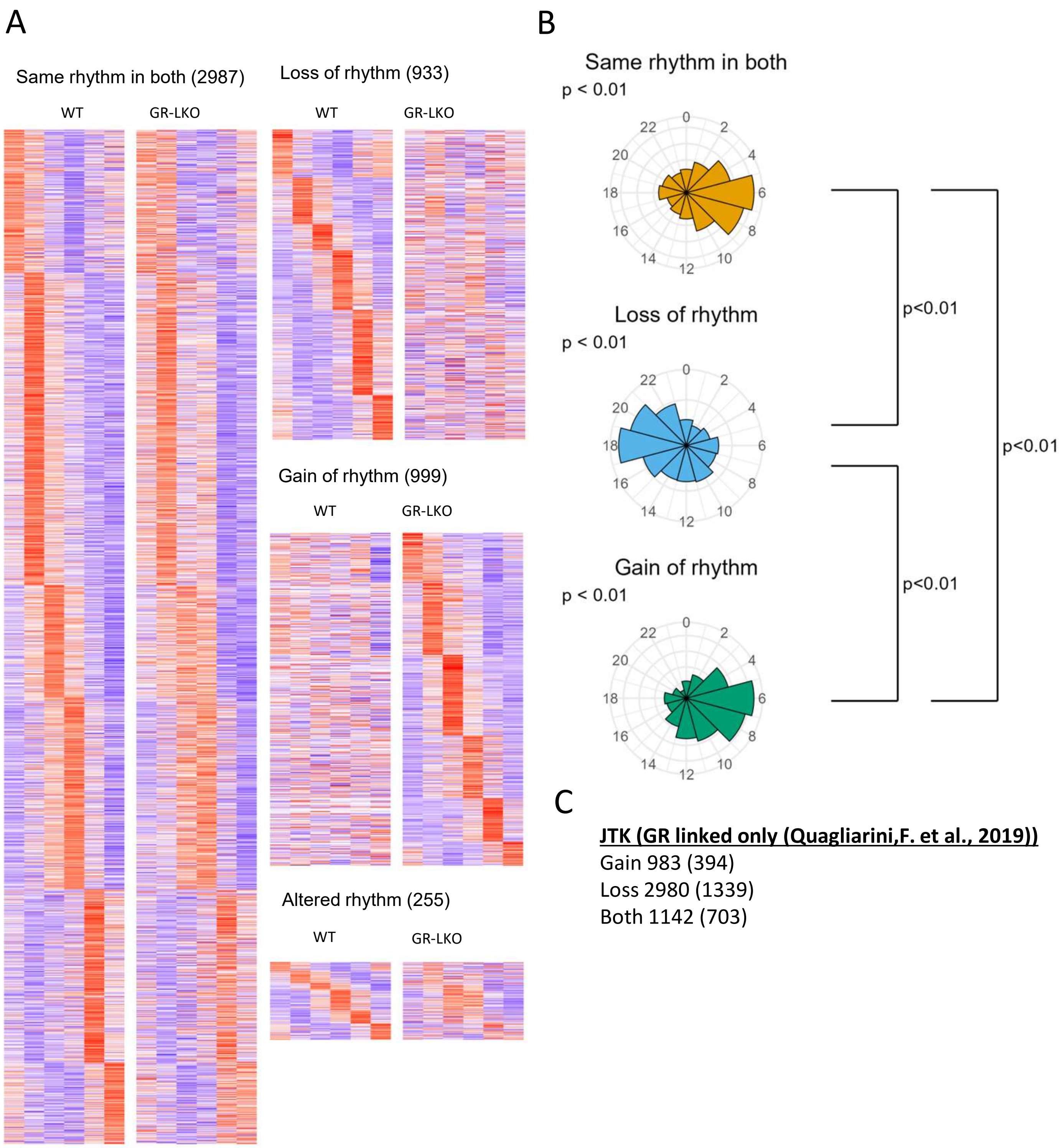

C

<u>JTK (GR linked only (Quagliarini,F. et al., 2019))</u>
Gain 983 (394)
Loss 2980 (1339)
Both 1142 (703)

Figure S6. Rhythmicity analysis of previously published rhythmic gene expression data in mouse liver (59).

A. CompareRhythms on previously published rhythmic gene expression data in mouse liver (59). Heatmaps showing z-score normalised gene expression in the 4 rhythmic groups. Ordered by phase of gene (in relevant condition and WT in altered rhythm). B. Peak phase of genes by rhythmic group; same rhythm in both (phase of shared signal), gain of rhythm in GR-LKO (phase in GR-LKO) and loss of rhythm in GR-LKO (phase in WT). C. Results from JTK cycle analysis of previously published data (36). In their paper only GR linked genes are presented, these numbers are in brackets.
